# Marital Status, Living Arrangements and Mortality at Older Ages in Chile, 2004–2016

**DOI:** 10.3390/ijerph192113733

**Published:** 2022-10-22

**Authors:** Moisés H. Sandoval, Marcela E. Alvear Portaccio

**Affiliations:** 1 Institute of Nutrition and Food Technology (INTA), University of Chile, Macul 7830490, Chile; 2 National University of Colombia, Bogotá 111321, Colombia

**Keywords:** marital status, living arrangement, older adults, mortality, Chile

## Abstract

The risk of mortality in old age is associated with marital status and living arrangements. There is still little knowledge about this in Latin America. Our objectives are to examine the association between marital status, living arrangements and mortality of older adults (>60 years) in Chile, and to test whether this association varies when demographic, socioeconomic and health factors are included. We used data from the Social Protection Survey, and mortality data were linked to the Civil Registry. We estimate a series of Poisson regression models. Our results show a clear association between marriage and longevity, since even controlling for demographic, socioeconomic and health factors, we found that separated or divorced, widowed, and unmarried people showed higher relative mortality compared to married people (IRR1.24, IRR1.33, IRR1.35, respectively). Considering only living arrangements, the results show that living alone, alone with children, with children and other relatives or in other arrangements is associated with higher mortality (IRR1.22, IRR1.27, IRR1.35, IRR1.35, respectively) compared to those living with their partners and children. However, considering marital status and living arrangements together, we find that survival among older adults was strongly associated with marital status. Marital status continues to be a direct measure of living arrangements among older adults in Chile.

## 1. Introduction

The positive influence of social relations on health and mortality has been well documented, mainly through the study of structural social relationships such as marital status, living arrangements and cohabitation in developed countries and more recently in some Asian countries [1,2,3,4,5]. The protective effect of marriage on longevity acts through the mechanisms of integration, support (emotional, economic), regulation and social control (theory of social causality) [1,2,6] and/or through direct and indirect marital selection (selection theory) [6,7,8,9,10,11]. Previous studies have consistently shown that mortality evolution has been more favorable among married people [1,5,7,8,10,11,12,13,14,15,16,17,18,19]. It has been reported that the mortality of unmarried people is higher compared to married people. However, after disaggregating the group of the unmarried (e.g., into never married, widowed or divorced and/or separated people) the results have been inconclusive. Some argue that it is divorced and/or separated people who present the greatest risks of mortality compared to married people [6,7,8,12,14,18], while others point out that it is the widowed who show the highest mortality [10].

Studies analyzing the influence of marriage on mortality by sex suggest that the protective effect is beneficial mainly for men [7,8,10,11,13,17,18,20,21,22,23,24,25,26,27]. It has been highlighted that the effect of marital status on mortality decreases as age increases [17]. Finally, findings in a good number of countries suggest that marriage protection mechanisms work in various social settings [8,12,14,18].

Demographic transformations such as a decrease in fertility and mortality and an increase in life expectancy have caused substantial changes in household formation in recent decades. There have also been changes in the older population, for example, the increase in consensual unions, separations, and divorces [18,25,28]. This has moved the focus of the study towards the role of living arrangements as a more appropriate predictor of social support, and therefore of the risk of mortality among the elderly population. Living arrangements would reflect the real, concrete availability of support and care available to elderly persons better than civil status, which only indicates someone’s marital status, but not if that person lives alone, with others, in an institutional home, etc. [29], thus weakening its value as a predictor variable of social support to the extent that it has ceased to be an indirect indicator of living arrangement in these new demographic contexts [25,29,30].

Living arrangements usually refer to the social environment of people, specifically to the structure of the home of the elderly person, indicating the people with whom the elderly person lives, whether they are family members such as the partner, partner and children, alone or in a home with another type of arrangement [31]. Considering that older people spend more time in the home than the other age groups [32], the living arrangement better represents the effective emotional, instrumental and material support for this population subgroup as the need for them increases with age. The living arrangement acts as the natural space in which informal transfers of care to and from older adults take place. Support that requires physical proximity—assistance in functional or instrumental activities of daily living—can be received when co-residing with someone, but mainly if it is the spouse/partner/children who are the most available caregivers, which favors survival and minimizes to some extent the demand for formal care [33].

Therefore, the living arrangement is assimilated in a proxy indicator of social bonding, of the availability of daily support within the household [17,25,34,35] and of intra- and intergenerational solidarity. The configuration of living arrangements responds to a form of social organization that is the result of a historical process and the social relations that make it possible; important differences can be observed in them and what they represent in each society. The living arrangement of older adults within multigenerational households is not predominant in Europe and the United States of America (USA), while in Latin America it continues to be so despite the significant increase in single-person households.

In line with the above, during the last decades, considerable evidence has shown that mortality risks at older ages are associated with living arrangements and that there is significantly greater mortality among those who live alone compared to those who live with at least one other person. Living alone with children or living with others is associated for both men and women with significantly higher mortality risks compared to those living with a spouse or partner. Living arrangements are much more beneficial for men, mainly living with their partner [17,18,23,25,29,34,35,36,37,38,39,40].

Some studies have jointly addressed marital status and living arrangements and their relationship to mortality at older ages, suggesting that this estimates the variation in mortality risk in old age better than if marital status or living arrangements are only observed independently [6,17,18,29,34,35]. The results of these studies have shown that living arrangement is a stronger predictor of mortality than marital status [17,29,34]. This would be an indication that the living arrangement better reflects the effective support that an older adult receives within the home.

Studies related to the association between marital status, living arrangement and mortality have an important tradition in Europe, the USA and more recently in some Asian countries, societies where the social, economic, and cultural codes surrounding the family and social institutionality of the older adult population are different. This association has been little explored in Latin America, despite the accelerated and heterogeneous aging process it is going through. We believe that the gap that still exists in Latin America in this line of study may be due to the absence and inconsistency of the information gathered from traditional data sources—censuses and vital statistics—and the incipient collection of longitudinal data.

We consider that our study of Chile can contribute to filling the existing gap in the region on the understanding of the association between structural social relations and mortality in the older population.

### 1.1. Population Aging and the Transformation of Marriage Patterns and Living Arrangements in Chile

Chile, like other Latin American countries, has experienced an accelerated process of population aging, characterized by declining mortality and fertility. Between 1950 and 2020, the infant mortality rate fell from 120 to 6 (per 1000 live births), while the average number of children per woman fell from 5.0 to 1.5 in the same period. The Chilean population has benefited from an impressive increased lifespan. Whereas a Chilean in the mid- 20th century expected to live 54.9 years, today life expectancy at birth for both sexes is 80 years [41,42]. This has contributed to the increase in the proportion of older adults from 5.4% to 18.1% between 1952 and 2022 [43,44], which is expected to reach 32.1% by 2050 [45].

Marriage was the norm in Chile until the late 1960s, and consensual union was the exception or a marginal phenomenon in the countries of the Southern Cone, as a result of a differentiated colonization process -countries with a small indigenous or Afro-American population- and greater European influence in relation to the rest of the countries of Latin America [46]. Free union has expanded since the last decades of the 20th century to the point of displacing marriage and remains increasingly a durable form of cohabitation [47]. According to the National Socioeconomic Characterization Survey CASEN 2017 [48], among those with partners in the population (formally married, cohabiting) aged 60 and over, 87.4% are formally married and 12.8% are cohabiting, while among the population aged 15–59, 58.8% are formally married and 41.1% are cohabiting. Although we do not have information on how the population aged 60 years and over who are now widowed constituted their unions, nor do we have information on the proportion of the population aged 15 to 59 years who are formally married, it is no less true that the proportion of this age group whose union is cohabitation is striking, which indicates the transformation in the constitution of unions in Chile.

In the rest of Latin America, consensual unions are the predominant form of union in most countries; this has been the case since colonial times and has expanded and deepened in recent decades in countries that had low levels of consensual unions, including Chile [49,50]. The connotation of the living arrangement in Latin America is based on the fact that it is the main form of support provided by families to their elderly parents or older relatives. Intergenerational co-residence is a tradition and a way of ensuring that the elderly are cared for by their families, mainly because there is an incongruence between the social and institutional conditions in the region and the process of population aging, and family transferences are the main source of support for the elderly population [31]. The region is traditionally characterized by affective links between generations, so families are the first to receive the impact of population aging [51]. In contrast to countries in Europe and the USA where there is an institutional construct—pensions, transfers, care centers—which have somewhat replaced the need for the family, households are the main providers of transferences in Latin America. According to United Nations 2019 [52], although there was a significant increase in the proportion of single-person households among people over 60 years of age from 5.4% in 1970 to 11.6% in 2002, many people over 60 years of age lived in households with extended families (43.2%) in 2002. 

Although Chile is at an advanced stage of population aging, the change in the pattern of unions has been slower, and among older cohorts, marital status is still a direct indicator of living arrangements. However, the data point to a transformation of unions towards the Latin American pattern—consensual unions—as predominant, thus living arrangement as a unit of analysis will gain relevance for understanding the factors associated with mortality among the older adult population in Chile.

### 1.2. Aims and Hypotheses

The objectives of this study are:

1. To examine the association between marital status, living arrangements and mortality in the older adult population.
2. To examine the degree to which the association between marital status, living arrangements and mortality is moderated by socioeconomic factors and health behavior of the elderly.
3. To examine the joint role of marital status and living arrangements on the mortality of the older adult population.

Our working hypotheses are:

**Hypothesis** **1** **(H1).** *Married people have lower relative risks of mortality compared to unmarried people (divorced, widowed and never married). This differential is constant even after controlling for socioeconomic and health factors.*

**Hypothesis** **2** **(H2).** *Older people who live with their partners or spouses have lower mortality compared to those who live alone or in other types of living arrangements.*

**Hypothesis** **3** **(H3).** *Marital status is a better predictor of mortality than living arrangements at an older age.*

## 2. Materials and Methods

The data are from the “Encuesta de Protección Social” (EPS). The EPS is the largest longitudinal survey in the country (around 16,000 people over 18 years of age are followed). Since 2004 (second survey) the sample is representative nationally, therefore the 2004 survey forms our baseline data. The information captured is of high quality and is commonly used to design and evaluate public and pension policies in the country. The EPS was designed initially to link information from administrative records [53], due to which it is a valuable questionnaire and a source of data, which is still rare in Latin America. The mortality data were obtained by linking the 2004 EPS with the vital statistics of the Civil Registry of Chile, which minimizes possible measurement errors. The database with the link between records (anonymized) was provided by the Subsecretaria de Prevision Social de Chile (Undersecretariat of Social Welfare of Chile). Therefore, this study was based on secondary analyses and did not need to be submitted to the Scientific Ethics Committee.

We included all adults above 60 years of age at the time of the 2004 survey, 3647 people. The total sample under analysis has information on each of the variables under analysis. Therefore, no statistical imputation was necessary for possible missing covariates. The follow-up period was from this moment until death, or if the person was still alive, to 31 December 2016. During this period 1480 elderly people died. 

### 2.1. Variables

The key independent variables are marital status and living arrangements. We used the information on members of the household of the interviewee, classifying marital status into four groups: (1) married or cohabiting; (2) separated or divorced (i.e., married at some point); (3) widowed; and (4) unmarried or never married; and seven types of arrangements: (1) living alone; (2) living with partner and children; (3) living only with the partner; (4) living only with children; (5) living with a partner and other family members; (6) living with children and other family members; and (7) other arrangements. The last category includes elderly people who live only with other family members and other non-relatives. It should be noted that only 172 (4.7%) older adults reported living together, so it was decided to consider one category for those who are married or cohabiting.

Age categories were 70 or less and more than 70, given the composition of the sample. Since 54% of the sample was between 60 and 69 years old, we opted to separate the sample into those under 70 and those over 70. The dichotomous sex variable was scored as 1 for men and 2 for women. 

We included education as a proxy of socioeconomic status, considering four groups: (1) 0–4 years; (2) 5–8 years; (3) 9–12 years; and (4) 13 or more years; and social participation as a proxy of social networks. The latter is a dummy variable (0 no participation, 1 participation), including participation in different types of organizations (religious, syndicates, social, cultural, etc.).

We also included aspects of health behavior and health status as covariates. The EPS of 2004 asked how often the person played a sport or did physical activity, with eight categories of responses: (a) every day; (b) 5–6 days per week; (c) 3–4 times per week; (d) 1–2 times per week; (e) 1–3 times per month; (f) a few times in a year; (g) never or almost never; and (h) does not know/does not answer. We grouped these into three categories: (1) does not exercise (f, g, and h); (2) low frequency of exercise (d, and e); and (3) frequent exercise (a, b, and c). We included the variable “functional limitations that limit activities of daily life (ADL)” as a proxy for health status. This variable was divided into two groups; (1) No; (2) Yes for one or two basic activities of daily life; and (3) three or more limitations.

Finally, it should be noted that except for age, the variables used in this study are fixed or stable, i.e., they are the values observed in the 2004 questionnaire used as the baseline. The covariates selected were the dimensions commonly examined in the literature for this type of study.

### 2.2. Analytical Strategy

Poisson regression models are a type of survival model that estimate the number of deaths during the follow-up period as a function of age and person-years of exposure [54]. This type of model has been widely used in the study of mortality differentials [55,56,57,58]. We modeled the number of deaths during the observation period as a function of person-years of exposure, marital status, living arrangements, socioeconomic conditions, health behavior and health status of the elderly. The basic model is:

$$\log\;\left(deaths\right)=\beta_{\;}*X_{i}+\log\;\left(Exposure\right)$$

*X* is a vector of variables, and the coefficient *β* is estimated by maximum likelihood. Each of the models was controlled for sex, age, time of exposition and risk of death measured as the number of person-years. The first series of models includes the key independent variable “marital status.” The first model of this series measures the effect of marital status itself on the mortality of the elderly.

Model 2 adds the demographic controls of sex and age. Model 3 adds socioeconomic conditions, specifically education. Model 4 incorporates the social participation variable as a proxy for social networks. Model 5 adds the variable of health behavior (physical activity). Finally, Model 6 adds the variable limitation in ADL.

In the second series of regression models, the variable “marital status” is excluded and living arrangement is added. Covariables were added in the same order described above. Finally, two additional models were used, including living arrangement and marital status, to establish which is a better predictor of mortality among the elderly population. Other models (not reported) were also estimated to examine the interaction between marital status and living arrangements. However, the results were not significant and did not provide relevant evidence for the analysis.

It is pertinent to clarify that the incorporation of the covariates in the models was undertaken according to the factor to which they belong. We first examined the pure association between marital status or living arrangement and mortality. Next, we incorporated the demographic variables sex and age. Third, we incorporated schooling as a proxy for socioeconomic status. Then, we added the social participation variable, followed by the physical activity variable as a proxy for health behavior. Finally, we added the functional limitations variable as a proxy for health status. In other words, we considered an additive strategy of the factors analyzed. The results of the Poisson regression models are presented as Incidence Rate Ratio (IRR). Due to the complexity of the EPS sample design, all estimates were population weighted and standard errors were adjusted for complex survey design using the svy commands. All the analyses were performed with Stata software, version 16 (StataCorp, College Station, TX, USA).

## 3. Results

Table 1 presents the descriptive analysis of the sample of 3647 elderly people, equivalent to 38,160 person-years. During the observation period, 1480 of them died. In total, 10.2% of the elderly lived alone in 2004, while 17.4% lived with a partner and children (nuclear family), 15.9% lived alone with a partner or spouse, and 5.6% lived with children. The highest proportion of older adults (27.5%) lived with partners and other family members, while 10.9% lived in another type of arrangement. It should be highlighted that 58% of older adults declared being married, 6.5% separated or divorced and 23% widowed. That is, about 90% of older adults are married or were married at some point in their lives. Only 12.5% reported being unmarried. Those who lived alone were mostly widowed and unmarried people, while those who live with their children, or with children and other relatives, were mostly widowed or separated older adults. Finally, mostly older adults who never married were living with relatives or non-relatives (other arrangements).

There were 49.3% men and 50.7% women in the sample; 46.4% of the elderly were 70 or older in 2004. The sample analyzed included mostly older adults with low education (72.7% had eight or fewer years of education) who do not participate in social life (68.7%), do not perform physical activity (85.8%) and declared that they have no limitations in carrying out basic activities of daily life (79.6%).

Table 2 presents the Incidence Rate Ratio of the association between marital status and mortality in the elderly in Chile for each of the six estimated regression models. Model 1 permits visualizing the pure effect of “marital status”. The results show that being widowed or unmarried is associated with higher mortality at 47.9% and 16.8%, respectively, compared to those who are married. In Model 2, controlling for sex and age, we observed the expected increase in the relative risk of death for widowed and unmarried people compared to the reference group. The relative mortality was higher for men and increased with age.

Adding education in Model 3 as a proxy for socioeconomic status produced a decrease in the size of the effect of marital status on the mortality of widowed people, maintaining the size among unmarried people. The existence of an educational gradient in mortality at older ages is confirmed; those with less schooling had a relative mortality 36% higher compared to more-schooled older people. The results of incorporating social participation (Model 4) show that the size of the effect of marital status is quite similar to that found in Model 3. However, having an active social life had a protective effect of 22% on mortality.

Model 5, adding the physical activity variable, showed that the size of the marital status effect remained practically invariable as in Model 4. However, highly frequent physical activity was associated with a 32.9% lower relative mortality among married older adults in comparison to not doing physical activity. 

Finally, Model 6 added the limitations variable as a proxy for health status, showing that although health status is a good determinant of mortality, marriage continued to be a protective effect for survival at older ages. In fact, older people who never married, even controlling for demographic, socioeconomic and health variables, had 36% higher mortality (*p*-value < 0.001) in comparison to married people, while those older adults who suffered the death of their spouse had a 34% greater relative risk of death (*p*-value < 0.001) compared to those who are still married. 

In Table 3 are the IRR of the association between living arrangements and mortality. Model 1 shows that older adults who live alone, those who live in other living arrangements and those who live with children and other family members have 35%, 34.9% and 36.1% greater mortality rates, respectively, than those who live with partners and children (reference group). In Model 2, considering sex and age, the results are like those described in Table 2. That is, mortality is higher in men, and it increases with age. However, when each subcategory of living arrangement is reviewed, it is observed that there is an increased relative risk of death for those living in an arrangement in which the spouse or partner is not present.

Adding education into Model 3, there was a decrease in the size of the effect of residence arrangement over mortality. However, the trend remains like that observed in the model, meaning that living in a residential arrangement in which the spouse or partner is not present is indicative of an increased relative risk of death. As in the set of previous models (Table 2), an educational gradient is observed among the older adult population, which is shown by the fact that those highly educated have a mortality rate 37.5% lower than those less schooled.

When social participation is considered in Model 4, and incorporating physical activity in Model 5, the effect of the living arrangement on mortality remained almost identical, and as expected, maintaining an active social life and exercising acted as protectors against mortality at older ages. Finally, in Model 6, adding the limitation variable, a reduction in the size of the effect of the living arrangement on mortality was observed. In fact, only the category “other arrangements” was significant (*p*-value = 0.05), indicating that those older adults living with other members—family and non-family—had a 33% greater relative mortality compared to those living with their partners and children.

Table 4 presents the results of the models that included marital status and living arrangements. Because health status is a close and direct determinant of mortality, we estimated a model without the limitation in ADL, and a second model in which it was included.

Model 1 showed that combining both main independent variables, marital status continued to be associated with mortality at older ages, despite the decrease in the size and significance of the effect. It is evident that widowed people present a 38.3% higher relative mortality compared to married people, while unmarried people have a 29.5% higher mortality in relation to the same reference group. The living arrangement variable was most relevant when considered simultaneously with the marital state. 

Model 2, adding the limitations in AVD, showed that the effect of marital status declined substantially and became marginal or not significant. Even controlling for demographic, socioeconomic, health variables and living arrangements, being married was associated with 26.9% lower mortality than in widowed people (*p*-value = 0.05).

In summary, considering both independent variables together, our findings suggest that survival among older adults in Chile is more strongly associated with marital status than with living arrangements.

## 4. Discussion

### 4.1. Marital Status and Mortality at Older Ages

The results of our study confirm that marital status in Chile is associated with the mortality of the elderly population (H1), as has previously been noted in other countries [1,5,7,8,10,11,12,13,14,15,16,17,18,19]. Our findings suggest that the relative mortality is higher among widowed and never married people compared to married people, which is in line with previous evidence [6,8,10,11,12,13,14,17,18,30,35]. However, we did not detect significant differences between married and divorced or separated people, which has been suggested in other studies [6,8,12,13,14,17,18,25,35]. 

As expected, we found that with an increase in age there is a decrease in the effect of marital status on mortality, a result that is in line with the results of Staehelin et al. [17] for Switzerland and Herm et al. [29] for Belgium. Although gender differences were not explored in depth in this study, our findings coincide with existing evidence in the sense that the protective effects of marriage are more beneficial for men than for women [8,10,11,13,17,18,20,21,22,23,24,25,26,27].

We also found that being married is strongly associated with longevity, even after controlling for demographic, socioeconomic and health variables (Table 2). Surprisingly, when the functional limitations variable was added, the differential between married and widowed people was reduced, while the differential between married and unmarried people increased slightly (Models 5 and 6, Table 2). This suggests that unmarried people on average have a worse state of health (more functional limitations); controlling for all covariates, they showed a relative mortality 36% higher than married people.

Our results support the theory of marriage as a protective factor for survival in older ages. Although these findings are not surprising in the light of what has been described mainly for developed countries, it contributes to clarifying this phenomenon in the Latin American context, a region in which it has been scarcely studied, with exception of a Brazilian study [59] and Chile, where this is the first of its kind.

These results are important for Chile because they show that currently a significant proportion of the older adult population is demanding and receiving a set of resources for their care from the family, which explains their greater survival, understanding that formal marriage is the main legitimizer of this [60]. While recognizing the family’s protective function, this forces us to think about how to construct a public policy that, while respecting what the family represents culturally, also makes it possible to smooth the effects of time, poverty and health when the head of the family is the only or the most important support of care transferences for the older adult population.

The consistency of similar findings in different countries in social and cultural terms, even with variations in their pace of demographic transformations, suggests that the protection mechanisms of marriage work in different environments or contexts regardless of the cultural variations that exist in social norms related to marriage.

### 4.2. Living Arrangements and Mortality Risks

This study also found that the relative mortality in old age in Chile is also associated with living arrangements (H2), as previously shown by different authors [17,18,23,25,29,34,35,36,37,38,39,40]. Specifically, we found that the relative mortality is higher among those who live alone, live with other relatives and children or live in other arrangements, compared to the reference group of those living with a partner and children. These results coincide with what was reported by Li et al. [39] for China, and by Herm et al. [29] for Belgium.

The results show that the relative mortality of older people is higher in any living arrangement in which the spouse or partner is not included. It is mainly the figure of the spouse or partner that acts as a mediator of care and social support within the living arrangement. Cohabiting with a partner or spouse represents greater emotional benefits and the possibility of mutual care. Dependence on care is more constant and reliable if it is from the spouse, whereas living alone with children and other family members or residing in other arrangements represents greater relative mortality compared to living with the spouse.

It is worth highlighting that in our study most of the individuals who did not live alone lived with their spouses or partners; that is, intimate social relationships and those of proximity overlap, exerting a double protective effect. Although the association and significance of the living arrangement on mortality are maintained considering age and sex, the size of the effect weakened among the population over 70 living alone, coinciding with what was found by Herm et al. [29] and Poulain et al. [25], in the sense that as age increases, the risks of living alone become closer to those of living with a partner. In addition, it is important to highlight the fact that the relative death risk increased with age among older adults who live in other arrangements and with children and other family members. This may indicate that by requiring greater transfer of informal care—greater support as age increases—the situation worsens for elderly people who do not live with their partner or spouse. Although this study does not address gender differences, these results seem to support that this mostly benefits men, which is in line with what is described in other societies [17,25,35,39].

These results are the first to our knowledge in Latin America and Chile. Their importance lies in the fact that they confirm the existence of a traditional norm of the living arrangement as a central strategy to improve the general level of the older adult population in contexts of institutional fragility, where the mechanisms of social transfers are unstable, which is a constant in Latin America as already mentioned. This forces a discussion on what social pacts are needed and should be made in a context of weakening of marriage as a legitimizer of the family and the rise of consensual unions to deal with the best way to plan public policies with perspective generational equity, given the imminent growth of the population of older adults within the total population.

### 4.3. Marital Status, Living Arrangements, and Mortality at Older Ages in Chile 

This study adds to the work that has jointly explored both variables [17,18,25,29,34]. Our results suggest that in Chile marital status is a better predictor of mortality at older ages than living arrangements. These results contrast with findings in other societies [17,29,34]. This may be explained by the fact that marriage in older cohorts was the predominant pattern of unions, which indicates that marital status is still a direct indicator of living arrangements in Chile. This is corroborated by considering that 58% of the sample under analysis declared being married at the time of the survey. However, the effect of marital status on longevity is expected to decrease in the future, due to the transformation of marital union patterns observed since the 1970s [46]. In the coming decades, living arrangements will perhaps be a better predictor of mortality in old age, to the extent that marriage continues to be less frequent and cohabitation outside marriage becomes more frequent among new cohorts, as is observed in European countries [17,18,29,34].

### 4.4. Limitations

The main limitation of this study is that the information on marital status and living arrangements was the information obtained from the baseline and does not consider the changes that may have occurred over time in relation to marital status or the type of living arrangement. However, we believe that these results, even though they may be overestimating the effect of marriage, allow us to visualize that marriage in cohorts of older adults from conservative, developing societies such as Chile is closely linked to longevity. Secondly, it is known that older people who live in or moved to collective households (care institutions, homes, etc.) show higher levels of mortality compared to the general population [61]. However, this could not be examined since the source of data used here, as well as most of the surveys in the country, do not incorporate the institutionalized population. Despite this limitation, our results are clear in suggesting that for Chile, marital status is a better predictor of mortality at older ages. Although we found that marriage seems to be more beneficial to men than to women, this study did not delve into the analysis of gender differences associated with marital status, residence arrangement and mortality. This finding should encourage the development of future studies to go deeper into this issue.

Finally, in the context of the pandemic, it is very likely that there have been important changes in the dynamics of the living arrangements of the older adult population; however, we do not yet have sufficient, stable information to allow us to make any inference about this, and it would be desirable to be able to explore it.

## 5. Conclusions

This study showed evidence that marital status and living arrangements are important, independent predictors of mortality among the older adult population in Chile. However, when both variables are considered together, marital status is a better predictor of mortality than living arrangements. This may be attributed to the fact that marital status is a direct indicator of the living arrangements of these cohorts in Chile, mainly formal marriage. However, as the older cohorts (studied here) die, the association between marital status and mortality will cease to be so important, due to the changes that have been observed in the patterns of family formation in Chile. Thus, the living arrangement would be consolidated as the main key variable of structural social relations to understand its association with mortality in the older adult population. However, this hypothesis will have to be tested in the future.

Our study, being the first, fills the gap in the study of the relationship of marital status and living arrangements—independently and jointly—with mortality risks in Latin America in general and in Chile in particular. This study used a longitudinal database linked to demographic data from the civil registry, which makes the data used in this study exceptional, and in that sense, the research is innovative and makes a valuable contribution to the existing literature on the association between marital status and living arrangements and mortality in the older adult population, if we also take into account that longitudinal surveys in the region are still incipient.

The findings of this study provide valuable information for the development of evidence-based public policies aimed at the construction of public policy for old age, care and health of the older adult population in Chile, since the growth of the older adult population poses a challenge to society. It is necessary to define the social and institutional agreements that lead to the establishment of care distribution systems as a social responsibility of the State, families, and the market, the so-called social organization of care, so that the family is not the main unit responsible for the well-being of the older adult population as it has been until now, due to the high costs that this has on the life trajectories of women who are the main informal caregivers.

Finally, considering that 12% of the Chilean population reports belonging to an indigenous nation, it is pertinent that future studies examine whether marital status or living arrangements are associated with longevity according to ethnicity.

## Figures and Tables

**Table 1:** Description of the variables in the 2004 survey.

Variables	Alone (%)	With Partner and Children (%)	With Partner (%)	With Children (%)	Partner and Others (%)	Children and Family Member (%)	Other Members (%)	Total (%)
Living arrangement	10.2	17.4	15.9	5.6	27.5	12.5	10.9	100.0
Marital Status								
Married	3.5	93.6	93.6	4.4	92.9	3.1	3.5	58.0
separated-divorced	19.1	1.6	0.5	18.5	1.6	16.2	6.3	6.5
Widowed	46.1	3.9	5.2	68.3	4.7	65.0	32.3	23.0
Single	31.3	0.9	0.7	8.8	0.8	15.7	57.9	12.5
Sex								
Male	41.5	61.5	56.6	20.0	65.6	22.3	31.3	49.3
Female	58.5	38.5	43.4	80.0	34.4	77.7	68.7	50.7
Age								
60–69 years	43.7	67.5	51.9	48.3	59.3	40.9	45.6	53.6
70 or more years	56.3	32.5	48.1	51.7	40.7	59.1	54.4	46.4
Education								
0–4 years	46.9	31.4	40.8	41.0	41.3	48.6	40.9	40.9
5–8 years	28.3	34.0	30.1	34.6	33.4	32.8	27.3	31.8
9–12 years	20.0	25.5	21.3	18.1	20.6	16.6	21.5	21.0
13+ years	4.8	9.1	7.8	6.3	4.7	2.0	10.3	6.3
Social Participation								
No	68.5	68.4	65.9	70.7	69.2	68.7	70.7	68.7
Yes	31.5	31.6	34.1	29.3	30.8	31.3	29.3	31.3
Exercise								
No	87.2	83.1	84.6	87.3	86.9	87.9	84.1	85.8
Low Frequency	6.0	8.0	6.6	5.4	4.9	4.4	6.4	5.9
High Frequency	6.8	8.9	8.8	7.3	8.2	7.7	9.5	8.3
Functional Limitations								
No	80.3	88.1	78.4	74.6	79.9	70.9	79.0	79.6
Yes	19.7	11.9	21.6	25.4	20.1	29.1	21.0	20.4
Sample Size (N°)	371	636	578	205	1001	457	399	3647
Person Years (N°)	3759	6887	6272	2178	10,589	4602	3974	38,160
Deaths (N°)	167	221	233	77	400	204	178	1480

Source: Own elaboration based on EPS 2004.

**Table 2:** Rate ratios for the association between marital status and mortality in older adults, 2004-2016.

Variables	Model 1 IRR	Model 2 IRR	Model 3 IRR	Model 4 IRR	Model 5 IRR	Model 6 IRR
Marital Status						
Married (ref)	1.000	1.000	1.000	1.000	1.000	1.000
separated-divorced	1.208	1.208	1.225	1.199	1.210	1.256 ɫ
Widowed	1.349 ***	1.479 ***	1.415 ***	1.418 ***	1.411 ***	1.343 ***
Single	1.168 ɫ	1.341 ***	1.341 ***	1.326 ***	1.339 ***	1.359 ***
Sex						
Male (ref.)		1.000	1.000	1.000	1.000	1.000
Female		0.554 ***	0.561 ***	0.574 ***	0.565 ***	0.525 ***
Age						
60–69 years (ref.)		1.000	1.000	1.000	1.000	1.000
70 or more years		3.478 ***	3.400 ***	3.400 ***	3.415 ***	3.173 ***
Education						
0–4 years (ref.)			1.000	1.000	1.000	1.000
5–8 years			0.800 ***	0.802 ***	0.812 ***	0.862 **
9–12 years			0.711 ***	0.710 ***	0.730 ***	0.787 **
13+ years			0.640 ***	0.653 **	0.698 **	0.783 ɫ
Social Participation						
No (ref.)				1.000	1.000	1.000
Yes				0.776 ***	0.805 ***	0.808 **
Physical Activity						
No (ref.)					1.000	1.000
Low Frequency					0.794 ɫ	0.857
High Frequency					0.671 ***	0.730 **
Functional Limitations						
No (ref.)						1.000
Yes						2.164 ***

ɫ *p* < 0.1; * *p* < 0.05; ** *p* < 0.01; *** *p* < 0.001.

**Table 3:** Rate ratios for the association between living arrangement and mortality in older adults, 2004–2016.

Variables	Model 1 IRR	Model 2 IRR	Model 3 IRR	Model 4 IRR	Model 5 IRR	Model 6 IRR
Living arrangement						
With partner and children (ref.)	1.000	1.000	1.000	1.000	1.000	1.000
Alone	1.350 **	1.275 *	1.220 ɫ	1.227 ɫ	1.215 ɫ	1.148
With partner	1.143	1.013	0.997	1.000	0.999	0.907
With children	1.157	1.321 *	1.281 ɫ	1.266 ɫ	1.270 ɫ	1.115
Partner and others	1.136	1.015	0.980	0.981	0.976	0.902
Children and family member	1.361 **	1.435 ***	1.330 **	1.326 **	1.346 **	1.220
Other arrangements	1.349 **	1.384 **	1.367 **	1.352 **	1.338 **	1.329 *
Sex						
Male (ref.)		1.000	1.000	1.000	1.000	1.000
Female		0.565 ***	0.570 ***	0.584 ***	0.572 ***	0.526 ***
Age						
60–69 years (ref.)		1.000	1.000	1.000	1.000	1.000
70 or more years		3.520 ***	3.436 ***	3.437 ***	3.451 ***	3.201 ***
Education						
0–4 years (ref.)			1.000	1.000	1.000	1.000
5–8 years			0.802 ***	0.805 ***	0.816 ***	0.867 *
9–12 years			0.704 ***	0.702 ***	0.724 ***	0.781 **
13+ years			0.625 ***	0.638 ***	0.685 **	0.799 ɫ
Social Participation						
No (ref.)				1.000	1.000	1.000
Yes				0.780***	0.808 ***	0.811 **
Physical Activity						
No (ref.)					1.000	1.000
Low Frequency					0.791ɫ	0.854
High Frequency					0.667 ***	0.728 **
Functional Limitations						
No (ref.)						1.000
Yes						2.211 ***

ɫ *p* < 0.1; * *p* < 0.05; ** *p* < 0.01; *** *p* < 0.001.

**Table 4:** Rate ratios for the association between marital status, living arrangements and mortality in older adults, 2004–2016.

Variables	Model 1 IRR	Model 2 IRR
Marital Status		
Married (ref)	1.000	1.000
separated-divorced	1.193	1.189
Widowed	1.383 **	1.269 *
Single	1.295 ɫ	1.221
Living arrangement		
With partner and children (ref.)	1.000	1.000
Alone	0.860	0.956
With partner	0.893	0.905
With children	0.928	0.947
Partner and others	0.960	0.900
Children and family member	0.969	1.005
Other arrangements	0.944	1.105

Model 1 Control of sex, age, education, social participation, and physical activity. Model 2 Control of sex, age, education, social participation, physical activity and functional limitations. ɫ *p* < 0.1; * *p* < 0.05; ** *p* < 0.01; *** *p* < 0.001.

## Data Availability

The data presented in this study are available on the website of the Subsecretaría de Previsión Social, Gobierno de Chile (https://www.previsionsocial.gob.cl/, accessed on 4 April 2022).
